# Roux-en-Y Gastric Bypass Surgery Increases Respiratory Quotient and Energy Expenditure during Food Intake

**DOI:** 10.1371/journal.pone.0129784

**Published:** 2015-06-22

**Authors:** Malin Werling, Lars Fändriks, Torsten Olbers, Marco Bueter, Lars Sjöström, Hans Lönroth, Ville Wallenius, Kaj Stenlöf, Carel W. le Roux

**Affiliations:** 1 Department of Gastrosurgical Research and Education, Sahlgrenska academy, University of Gothenburg. Department of Surgery, Sahlgrenska University Hospital/Sahlgrenska, Gothenburg, Sweden; 2 Department of Surgery, Division of Visceral and Transplantation Surgery, University Hospital Zurich, Zurich, Switzerland; 3 Swedish Obese Subject’s Secretariat, Institute of Medicine, Sahlgrenska University Hospital, Gothenburg, Sweden; 4 Gothia Forum, Sahlgrenska University Hospital, Gothenburg, Sweden; 5 Diabetes Complications Research Centre, Conway Institute, University College Dublin, Dublin, Ireland; 6 Investigative Science, Imperial College London, London, United Kingdom; INRA, FRANCE

## Abstract

**Objective:**

The mechanisms determining long-term weight maintenance after Roux-en-Y gastric bypass (RYGB) remain unclear. Cross sectional studies have suggested that enhanced energy expenditure (EE) may play a significant role and the aim of this study was to reveal the impact of RYGB on each major component constituting total EE.

**Design:**

Six obese female subjects, without other co-morbidities, were assessed before and at 10 days, 3 and 20 months after RYGB. Indirect calorimetry in a metabolic chamber was used to assess 24h EE at each study visit. Other measurements included body composition by DEXA, gut hormone profiles and physical activity (PA) using high sensitivity accelerometers.

**Results:**

Median Body Mass Index decreased from 41.1 (range 39.1-44.8) at baseline to 28 kg/m^2^ (range 22.3-30.3) after 20 months (p<0.05). Lean tissue decreased from 55.9 (range 47.5-59.3) to 49.5 (range 41.1-54.9) kg and adipose tissue from 61 (range 56-64.6) to 27 (range 12-34.3) kg (both p<0.05). PA over 24h did not change after surgery whereas 24h EE and basal metabolic rate (BMR) decreased. EE after a standard meal increased after surgery when adjusted for total tissue (p<0.05). After an initial drop, RQ (respiratory quotient) had increased at 20 months, both as measured during 24h and after food intake (p<0.05).

**Conclusion:**

RYGB surgery up-regulates RQ and EE after food intake resulting in an increased contribution to total EE over 24h when corrected for total tissue.

## Introduction

Altered gut physiology after Roux-en-Y gastric bypass (RYGB) contributes to long-term weight loss that is associated with decreased morbidity and mortality in obese patients [1]. The expected total body weight loss is 25 to 35% within 10–14 months followed by weight stability for 1–3 years. Most patients gain a small amount of weight thereafter to level off around 20 to 30% below their pre-operative weight [1]. Weight loss following RYGB was thought to be mainly dependent on reduced energy intake, but not to any clinical significant degree to calorie malabsorption [2–8]. RYGB has also been postulated to increase energy expenditure (EE), thereby contributing to weight loss [9]. However, gastric bypass’s effects on energy expenditure in humans are controversial with some studies reporting increases and others no change or decreases of energy expenditure (EE) [10–12]. Discrepancies between human studies may be explained by heterogeneous patient populations and/or limitations of equipment used to determine EE and body composition. By using the “gold standards” indirect calorimetry over 24h in a “metabolic chamber” and dual energy X-ray absorptiometry (DEXA) as a measurement of body composition we have shown in a cross sectional study nine years after surgery that RYGB patients had higher 24h EE compared to weight loss matched VBG patients and that this was secondary to increased EE after food intake [9]. Similarly, Mingrone et al found increased EE after food intake in patients treated with biliopancreatic diversion [13]. These findings suggest that RYGB and biliopancreatic diversion are influencing the physiological basis for the thermic effect of food, presumably because the altered gastrointestinal anatomy influences the luminal digestion and mucosal exposure to the ingested nutrients.

The objective of the present study was to confirm and in further detail characterise meal associated thermogenesis (MAT) before and after RYGB using a state-of-the-art metabolic chamber. However, the “metabolic chamber” method assesses whole body thermogenesis being a compound variable of basal metabolic rate (BMR), muscular activity thermogenesis and MAT. Thus in order to accurately calculate MAT, the BMR and activity thermogenesis must be determined. BMR is defined as resting EE in the awake state. Muscle tissue has considerably higher EE compared to adipose tissue during resting conditions and is therefore the dominant determinant for BMR. Potential changes in BMR following weight loss must therefore be related to muscle mass rather than body weight demanding repeated high precision body composition assessments in longitudinal study settings. To what extent physical activity is influenced by bariatric surgery is controversial as self-reported and objective measures differ considerably [14–16]. In the present study we provided each patient, at every study visit, with four high sensitive accelerometers, one on each extremity. Recordings of spontaneous physical activity was useful as such, but also for standardisation of baseline EE before food intake, making it possible to compare repeated measurements of the thermogenesis induced by intake of a standard meal.

The primary aim of the present study was to investigate meal-associated thermogenesis at weight stability before and after RYGB within the same individual during 24 hours of the standardized conditions in the metabolic chamber. Secondary aims were to examine the surgical effect on 24h EE, BMR and spontaneous physical activity and the associated non-exercise activity thermogenesis (NEAT) during the weight loss phase as well as at weight stability.

## Methods

The study was performed in accordance with the Declaration of Helsinki and approved by the Regional Ethical Review Board in Gothenburg, Sweden (no:740–10). Study participants were fully informed and provided written consent. The investigation was performed at the Department of Gastrosurgical Research & Education, Sahlgrenska University hospital, Gothenburg, Sweden.

### Surgery

RYGB was performed laparoscopically as previously described [17]. The gastric bypass technique included a small gastric pouch (10–20 mL) connected to the jejunum in an antecolic-antegastric Roux-en-Y construction. The length of the Roux-limb was 75 cm and the entero-entero anastomosis was created 30 cm distal from the ligament of Treitz.

### The metabolic chamber

Energy expenditure was measured in an indirect calorimetry chamber [18] over 24h. The chamber was constructed as a small hotel room measuring 3x3x3 meters. Fresh, dried and filtrated air was circulated, while the temperature and humidity in the chamber was kept constant at 25°C and 40% relative humidity. Oxygen and carbon dioxide contents in the air leaving the chamber were measured constantly enabling an assessment of energy expenditure. A predefined protocol before each study visit was used to calibrate the chamber [18].

### Study participants

Six obese, female patients were included from the waiting list for RYGB. Patients were not prescribed any pre-surgical weight loss diet but were weight stable for at least three months prior to the study. They received similar multivitamin and mineral supplementations for one month before start of investigations. Patients were free from comorbidities, medication and were non-smokers. Study participants were invited to study visits during the same phase of the menstrual period.

### Blood samples

At arrival on each study visit, fasting blood samples were collected for plasma levels of free fatty acids, total cholesterols, high density lipoprotein (HDL), low density lipoprotein (LDL), iron, glycated haemoglobin (HbA1c), thyroid stimulating hormone (TSH), free thyroxin (fT4) and total triiodothyronine (T3), follicle stimulating hormone (FSH) and creatinine. As described above, at visit 1 & 4 and while still outside the chamber the patients were served a 400 kcal standard breakfast at 09.00 (8 E% protein, 36 E% carbohydrate, 56 E% fat) and blood samples were collected prior to and every 30 minutes for 150 minutes postprandially. Glucagon-like-peptide 1 (GLP-1), glucagon, oxyntomodulin and gastric inhibitory peptide (GIP) were assessed in these breakfast related samples.

### Protocol

The 24h energy expenditure was assessed four times: preoperatively (visit 1; weight stability), 10 days postoperatively (visit 2; start of weight loss), 3 months postoperatively (visit 3; weight loss phase), as well as after a self-reported three months period of weight stability after the surgical procedure (visit 4; weight stability). In total, patients spent 25 hours in the chamber at each visit. Thirty minutes in the beginning and 30 minutes at the end were excluded from analysis to allow for acclimatization, settling after entrance and preparation before exiting. Thus, 24 hours were available for analysis.

At arrival in the morning on each study visit weight and height were measured in light underwear. Body mass index (BMI) was calculated and Dual Energy X-Ray Absorptiometry (DEXA) (LUNAR Radiation, Madison, WI, USA) was used to assess total, adipose and lean tissue. The DEXA values were used in the analysis of energy expenditure and in adjustments relating recorded values to total or lean tissue.

Due to adaptation of the gut and small intestine after RYGB patients have a dramatically reduced food intake during the first months after operation. The test meals served at visit 2 and 3 during the weight loss phase, therefore contained a lower amount of calories compared to during visit 1 and 4 at weight stability.

**Fig 1:**
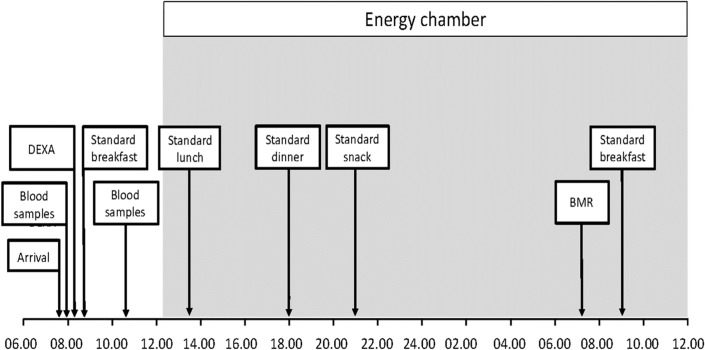
Protocol during study visits before as well as 10 days, 3 months and 20 months after gastric bypass surgery for six female study subjects.

Furthermore, in the evening before study visit 1 and 4, patients had a standardized dinner of mashed potatoes and meatballs at 19.00–20.00. Corresponding meals before visit 2 and 3 were semiliquid and contained 200 and 300 kcal respectively. The morning after these standard meals, the patient arrived to the laboratory at 07.30 after an 11h fast. Fig 1 shows the protocol during study visits.

#### Visit 1 & 4 (weight stability)

After sampling of fasting blood a standardised breakfast (400 kcal with 8 E% protein, 36 E% carbohydrate and 56 E% fat) was ingested outside of the chamber followed by a blood draw every 30 minutes for 150 minutes to profile responses of postprandial gastrointestinal hormones [9]. While in the chamber, the patients received four meals consisting of a 400 kcal fixed lunch at 13.30, a 600 kcal fixed dinner at 18.00, one 120 kcal fixed evening snack at 21.00 and a 400 kcal fixed breakfast the following morning at 08.40. The standard dinner consisted of meat balls and mashed potatoes (600 kcal with 13 E% protein, 41 E% carbohydrate, 46 E% fat) [9].

#### Visit 2 & 3 (weight loss phase associated with reduced ability to consume larger meals)

After a fasting blood test patients received a standardised liquid breakfast of 200 kcal. During the stay in the chamber at visit 2, standardized liquid meals containing 200 kcal each were served as lunch (13.30 h), dinner (18.00 h), evening snack (21.00 h) and as breakfast 08.40 h in the morning second day of the study visit. During visit 3 semiliquid meals were served in the chamber, containing 250 kcal at lunch (13.30 h), 300 kcal at dinner (18.00 h), 200 kcal as evening snack (21.00 h) and 300 kcal at breakfast 08.40 h in the morning of the second day of the study visit.

### 24 h energy expenditure and BMR

#### 24h energy expenditure (24EE)

24EE was expressed as kcal/24 hours. When appropriate, EE was expressed as mean calories per minute over the full 24h study period adjusted for total or lean tissue (kg) as assessed by DEXA for increased accuracy.

#### Basal metabolic rate (BMR)

Patients were woken up at 07.00 and allowed to go to the bathroom and were then instructed to get back into bed. From 07.30 to 08.30 patients were lying supine and awake in bed looking at the ceiling without detectable movements of the arms or legs. The EE recorded during the 30 minutes between 07.45 and 08.15 was used as BMR [19]. Already before entering the chamber, the patients had been carefully instructed both orally and in writing regarding the BMR recording routines in order to minimize potential disturbances due to, for example, unnecessary communication with staff.

### Physical activity

#### Measuring equipment

Body motion was measured during each visit in the energy chamber over 24 hours using three-axial accelerometers (ActiGraph GT3X+, Registered trademark by ActiGraph, Pensacola, Florida, USA, www.actigraphcorp.com) positioned on both wrists and ankles. The motility related raw-data were recorded in 1-second intervals (epoch 1s) from each arm and each leg separately, and were then summated into arbitrary activity units (AU) of the two arms and legs, respectively, averaged into 1 minute intervals (AU_1min_).

#### Analysis

The accelerometer-based assessments were separately validated against typical activities of daily living classified in metabolic equivalents (METS) [20, 21] (examples shown in S1 Table). Light intensity activity (watching TV, desktop work at the computer, household tasks with slow walking etc) corresponding to METS<2 were characterised by absence or very few leg (ankle) movements with ankle AU_1min_ ranging between 0 and 52, whereas the wrist AU_1min_ was much more variable ranging between 10 to 110. Moderate to intense activities (METS 3 to 6) with rapid walking, climbing and jumping resulted in ankle AU_1min_ 200 to 400, and wrist AU_1min_ 110 to 900 units. It was obvious that light intensity activity (METS<2) was best discriminated by the ankle recordings and was therefore defined as ankle AU_1min_ ≤50 units.

Total physical activity score was obtained by summation of wrist and ankle AU_1min_ and averaged over awake (08.30 to 22.00) and night time (22.00 to 07.00), thus excluding the period with morning activities before and the instructed rest, preceding the BMR assessment.

### Energy expenditure in relation to physical activity

The 1h period before the standardised dinner was used for detailed analysis of spontaneous physical activity and non-exercise activity thermogenesis (NEAT) being defined as the mean EE during the pre-meal hour minus the actual BMR. During the pre-meal period the subjects were awake and engaged in activities of daily living. The subjects were not instructed to restrict their physical activity but to avoid intentional exercise.

### Energy expenditure in relation to food intake

Meal associated thermogenesis (MAT) was assessed during the 150 min period after food intake. The subjects were instructed to stay awake, and were free to move around in the chamber. MAT was defined as the part of the recorded postprandial EE that exceeded BMR+NEAT but to make assessments comparable between study visits occasional 1 min intervals with ankle AU_1min_ ≥50 units (METS ≥2) were removed from analysis. As a rule at least 85% of the 1h pre-meal recordings had to be present in each individual to allow calculation of MAT. The meals at visit 1 & 4 (weight stability) were identical and allowed presentation and comparison of MAT in absolute terms. Due to the inability of patients to consume larger meals during visit 2 & 3, MAT had to be related to the ingested energy (MAT%) to allow comparisons between all visits. MAT% is similar to “diet induced thermogenesis” (DIT) as previously defined in the literature [22].

### Respiratory quotient

Respiratory quotient (RQ) was calculated from the ratio between total CO_2_ production and O_2_ consumed and was analysed for the 24h EE period, during the BMR recording as well as during the first postprandial hour.

### Statistical analyses

Median and range was used if not otherwise stated. In the figures boxplots and interquartiles are given. Differences between preoperative and postoperative values were analysed using Wilcoxon (pairwise), or Friedman test with Wilcoxon as post hoc contraster (>2 repeated measures). The trapezoid rule was used to calculate AUC. The conventional p<0.05 was used as the statistical rejection criterion. Prism 6 (GraphPad Softwar Inc) was used for statistical analyses. Power analysis made was based on data from previous study on bariatric patients using the same protocol and measuring equipment [9]. Power analysis to determine energy expenditure adjusted for total weight during 24 hours and after food intake gave a power exceeding 0.80 for a group of six subjects.

## Results

### BMI and body composition

Median BMI decreased significant from 41.4 kg/m^2^ preoperatively to 28 kg/m^2^ as measured 20 months after surgery (p<0.05). Lean tissue decreased significantly by 6.4 kg while the proportion lean tissue in relation to total mass increased significantly by 18.1% (both p<0.05). Adipose tissue decreased by 34 kg and the proportion adipose tissue in relation to total tissue decreased by 19.6% (both p<0.05). Table 1.

**Table 1:** Body composition in six females before and after gastric bypass surgery.

	Visit 1		Visit 2		Visit 3		Visit 4	
	Median	Range	Median	Range	Median	Range	Median	Range
Time after surgery	Preoperatively		9.8 days	(6.6 to 13.1)	2.8 months	(2.7 to 2.8)	20 months	17.7 to 22.3
BMI [kg/m^2^]	41.4	(39.1 to 44.8)	39.5 (*)	(38.3 to 42.4)	35.1 (*,#)	(28.5 to 36.8)	28 (*,#)	(22.3 to 30.3)
Total body mass [kg]	119	(111.4 to 124.1)	114.3 (*)	(107.3 to 117.5)	99.8 (*,#)	(74.9 to 105.4)	80.2 (*,#)	(63 to 83.1)
Lean tissue [kg]	55.9	(47.5 to 59.3)	52 (*)	(45.3 to 57.5)	50.1 (*,#)	(44.1 to 52.8)	49.5 (*)	(41.1 to 54.9)
Lean tissue/total body mass [%]	45.4	(42.6 to 50.2)	44.7	(42.2 to 50.2)	50.8 (*,#)	(48.1 to 58.9)	63.5 (*,#)	(52.8 to 76.9)
Adipose tissue [kg]	61	(56 to 64.6)	58.8 (*)	(54.3 to 62.7)	46.4 (*,#)	(28.2 to 52)	27 (*,#)	(12 to 34.3)
Adipose tissue/total body mass [%]	52.3	(47.5 to 55)	52.7	(47.5 to 55.4)	46.4 (*,#)	(37.6 to 49.4)	32.7 (*,#)	(19.1 to 44.1)
Excess BMI loss [%]			10.1	(6.2 to 13.1)	42.8 (#)	(29 to 79.2)	81 (#)	(67.7 to 113.6)
Total weight loss [kg %]			3.9	(2.2 to 5.3)	17.3 (#)	(11.5 to 32)	31.3 (#)	(26.8 to 50.2)

Median age at operation 41.1 years (Range 28.8 to 50).

Values are median (Range). Freidmans test with Wilcoxon as a post hoc contraster was used.

*; significant difference compared to visit 1; * = p<0.05.

#; significant difference compared to visit 2; # = p<0.05.

### Laboratory assessments

Gastric bypass surgery improved lipid profiles by increasing HDL (high density lipoproteins) and decreasing LDL (low density lipoproteins). Free fatty acids, iron, free T4, TSH, FSH and creatinine did not change and were within the reference range both before and after surgery. Haemoglobin, HbA1c and Total T3 decreased after surgery but remained within reference range. The area under the curve representing the postprandial profile for GLP-1, glucagon and oxyntomodulin were all elevated (all p<0.05). Postprandial levels of GIP were significantly lower after the operation (p<0.05). See Table 2 for details.

**Table 2:** Biochemical variables in six females before and 20.3 months after Roux-en-Y gastric bypass surgery.

	Reference range	Visit 1		Visit 2		Visit 3		Visit 4	
		Median	Range	Median	Range	Median	Range	Median	Range
**Fasting**									
Free fatty acid [mmol/L]	0.45 to 2.6	0.98	(0.56 to 1.6)	1.1	(0.85 to 1.6)	0.78 (#)	(0.73 to 1.5)	0.64	(0.45 to 1.1)
HDL [mmol/L]	1 to 2.7	1.35	(0.96 to 1.5)	1.2	(0.73 to 1.3)	1.25	(0.82 to 1.4)	1.95 (*,#,¤)	(1.5 to 2.2)
LDL [mmol/L]	1.4 to 4.7	3.45	(2 to 5)	2.75	(1.9 to 5)	2.35	(2 to 3.2)	2.4 (*)	(1.8 to 3)
Iron [µmol/L]	9 to 34	15	(12 to 20)	12.5	(11 to 22)	15.5	(12 to 20)	18	(7 to 26)
Hb [g/L]	117 to 153	140	(124 to 150)	140	(117 to 146)	131.5	(122 to 142)	125.5 (*,#)	(100 to 133)
HbA1c [mmol/mol]	31 to 46	35.5	(33 to 37)	34	(31 to 35)	33 (*)	(30 to 35)	33.5	(31 to 37)
Free T4 [pmol/L]	12 to 22	13	(13 to 15)	16	(13 to 19)	14	(13 to 15)	14	(13 to 15)
Total T3 [nmol/L]	1.3 to 3.1	2.05	(1.3 to 2.2)	1.83	(1.5 to 1.9)	1.75	(1.5 to 1.9)	1.45 (*,¤)	(1.2 to 1.6)
TSH [mIU/L]	0.3 to 4.2	1.95	(1.7 to 4.9)	2.15	(1.3 to 4.7)	2.5	(1.2 to 4.1)	2.05	(1.2 to 3.4)
FSH [IU/L]		6.75	(4.1 to 23.3)	6.06	(3.9 to 8)	6.75	(3.95 to 46.7)	5.37	(4 to 46.7)
Creatinine [µmol/L]	45 to 90	73.5	(64 to 87)	73	(70 to 76)	72	(56 to 87)	70	(59 to 79)
**After a 400 kcal standard meal**									
GLP-1 [pg/mL/min, AUC]		27	(18 to 31)					32 (*)	(22 to 38)
Glucagon [pg/mL/min, AUC]		172	(113 to 269)					825 (*)	(489 to 1718)
Oxyntomodulin [pg/mL/min, AUC]		142	(109 to 199)					713 (*)	(426 to 2266)
GIP [pg/mL/min, AUC]		427	(220 to 747)					248 (*)	(186 to 400)

HDL = High-density lipoprotein. LDL = Low density lipoprotein. HbA1c = Glycated hemoglobin. TSH = Tyroid-stimulating hormone.

GLP-1 = Glucagon-like peptide 1. GIP = Gastric inhibitory peptide.

AUC values for GLP-1, GIP, Glucagon and Oxyntomodulin were calculated using fasting and postprandial samples obtained every 30 minutes during 150 minutes following a standard 400 kcal meal.

Values are median (Range). Freidmans test with Wilcoxon as a post hoc contraster was used. AUC = area under the curve.

*; significant difference compared to visit 1; * = p<0.05.

#; significant difference compared to visit 2; # = p<0.05.

¤; significant difference compared to visit 3; ¤ = p<0.05.

### 24 h energy expenditure and BMR

BMR decreased after surgery, but there was no difference between visits after adjustment for total tissue weight, nor for lean tissue weight (Table 3). Absolute 24h EE decreased 20 months after surgery from 1.6 to 1.2 kcal/min (p<0.05), while 24h EE adjusted for total tissue weight increased from 12.9 cal/min/kg to 14.7 cal/min/kg (p<0.05) as shown in Table 3.

**Table 3:** Energy expenditure (EE) and respiratory quotient (RQ) in sex females before and after gastric bypass surgery.

	Visit 1		Visit 2		Visit 3		Visit 4	
	Median	Range	Median	Range	Median	Range	Median	Range
**Total 24 hour**								
EE; kcal/min	1.6	(1.3 to 1.8)	1.4 (*)	(1.3 to 1.6)	1.3 (*,#)	(1.1 to 1.3)	1.2 (*,#)	(1 to 1.4)
EE; cal/min/kg total tissue	12.9	(11.5 to 14.7)	11.8	(11.3 to 14.3)	12.9	(12.1 to 14.9)	14.7 (*,#)	(12.4 to 18.8)
RQ	0.76	(0.73 to 0.8)	0.71	(0.67 to 0.74)	0.72 (*)	(0.7 to 0.75)	0.83 (*,#,¤)	(0.76 to 0.87)
**BMR, measured awake in lying position, between 07.45 and 08.15 h**									
EE; kcal/min	1.2	(0.9 to 1.3)	1.1	(1 to 1.3)	1	(0.9 to 1.1)	0.8 (*,#,¤)	(0.7 to 1
EE; cal/min/kg total tissue	9.8	(8.5 to 10.8)	9.5	(8.8 to 11.7)	10.5	(9.7 to 11.9)	11.1	(8.7 to 1)
EE; cal/min/kg lean tissue	20.6	(19.5 to 23.6)	21.7	(19.8 to 23.3)	20.3	(19.8 to 21.3)	17.2	(13.9 to 20.7)
RQ	0.76	(0.67 to 0.85)	0.68	(0.62 to 0.71)	0.67	(0.65 to 0.69)	0.91	(0.66 to 1.05)
**During 1 hour before standard meal**								
EE; kcal/min	1.6	(1.5 to 1.7)	1.4	(1.0 to 1.7)	1.2 (*)	(1 to 1.5)	1.1 (*)	(0.8 to 1.4)
EE; cal/min/kg total tissue	13.6	(11.9 to 14.4)	12	(8.7 to 14.4)	12.1	(10.3 to 15)	15.4	(10.8 to 17.9)
NEAT; kcal/min	0.4	(0.3 to 0.6)	0.3	(0 to 0.4)	0.2	(0 to 0.5)	0.3	(0 to 0.6)
NEAT_METS≤2_; kcal/min	0.4	(0.3 to 0.6)	0.3	(0 to 0.4)	0.2	(0 to 0.5)	0.3	(0 to 0.5)
RQ	0.73	(0.72 to 0.78)	0.71	(0.67 to 0.77)	0.71	(0.67 to 0.74)	0.79	(0.70 to 0.95)
**During first postprandial hour between 18.20 and 19.20 h, after standard meal**								
Total energy expenditure and RQ								
EE; kcal/min	1.9	(1.5 to 2.1)	1.7	(1.5 to 1.9)	1.5 (*)	(1.4 to 1.7)	1.6 (*)	(1.4 to 1.8)
EE; cal/min/kg total tissue	16.2	(13.2 to 18.1)	14.8	(12.6 to 16.9)	15.8	(14.2 to 18.2)	20.6 (*,#)	(17.9 to 24.7)
RQ	0.8	(0.78 to 0.84)	0.78 (*)	(0.73 to 0.8)	0.8	(0.75 to 0.83)	0.87 (*,#,¤)	(0.84 to 0.89)
MAT: energy expenditure exceeding BMR and NEAT_METS≤2_								
MAT; kcal/min	0.4	(-0.1 to 0.5)					0.4	(0.4 to 0.6)
MAT; cal/min/kg total tissue	3.3	(-0.9 to 4.5)					5.8 (*)	(4.7 to 7.3)
MAT%: rise in energy expenditure after food intake in relation to calories ingested								
MAT%	0.1	(-0.02 to 0.1)	0.2 (*)	(0.1 to 0.7)	0.1 (#)	(0.002 to 0.2)	0.1 (#)	(0.1 to 0.1)
MAT%/kg total tissue	0.6	(-0.2 to 0.8)	1.6 (*)	(1.1 to 5.8)	1.4	(0.02 to 2.3)	1 (*,#)	(0.8 to 1.5)

Values are median (Range). Freidmans test with Wilcoxon as a post hoc contraster was used.

*; significant difference compared to visit 1; * = p<0.05.

#; significant difference compared to visit 2; # = p<0.05.

¤; significant difference compared to visit 3; ¤ = p<0.05.

### Total physical activity score and NEAT

**Fig 2:**
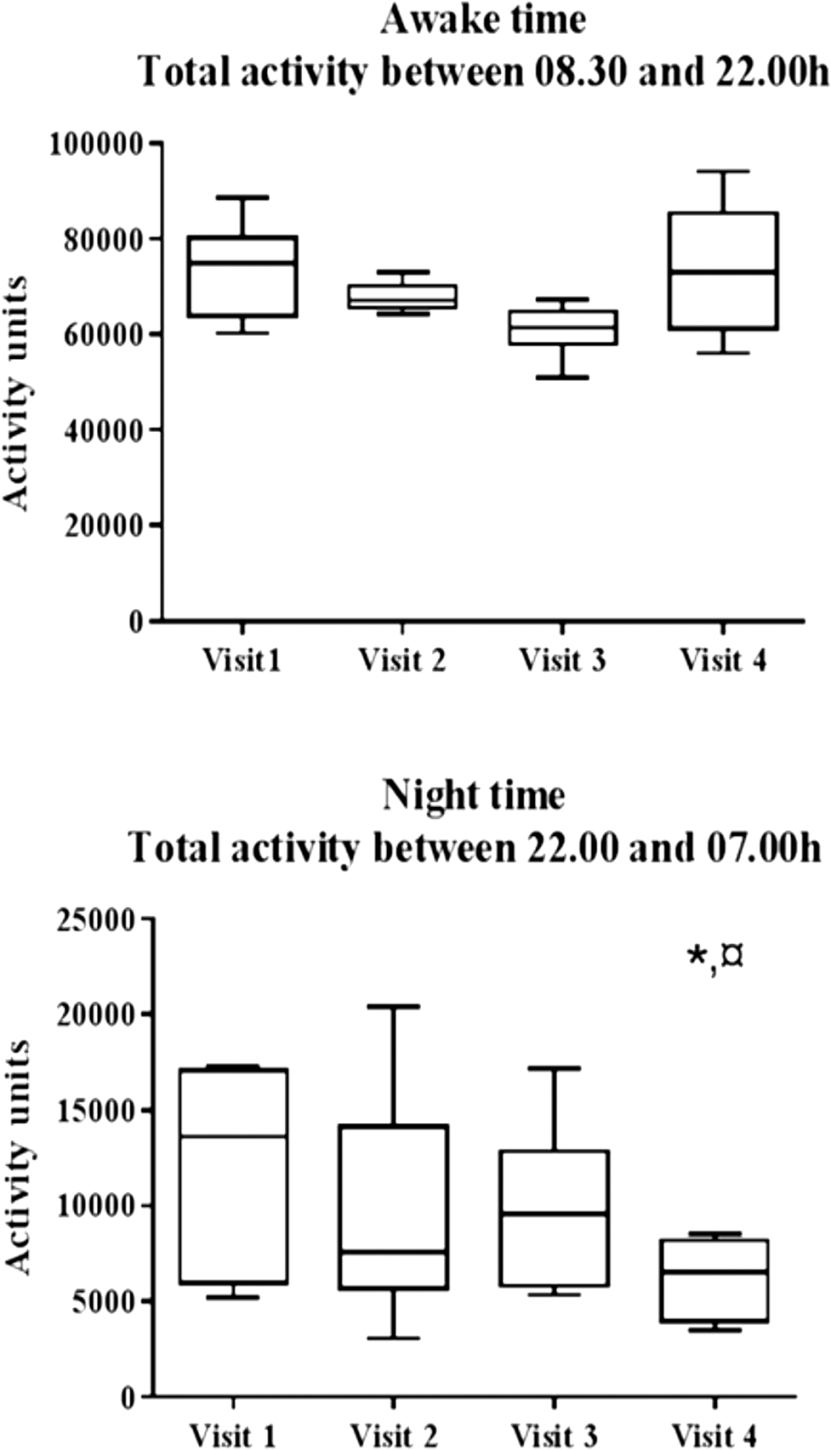
Total physical activity during awake time and during night time analyzed by three-axial accelerometers (Actigraph GT3X+) in six female subjects before and during weight loss (10 days and 3 months postoperatively) and at weight stability 20 months after gastric bypass surgery. *; significant difference to visit 1; * = p<0.05. ¤; significant difference to visit 3; ¤ = p<0.05.

The spontaneous (non-exercise) physical activity when awake as assessed by the four accelerometers did not change after surgery (Fig 2, upper panel). Night-time activity was very low and decreased significantly at visit 4 as compared to at the preoperative visit 1, p<0.05 (Fig 2 lower panel). NEAT was assessed during 1 h before dinner (see Fig 1 = study day protocol). Generally the physical activity level of the participants was low and none of them exercised in the chamber. As expected pre-prandial EE decreased with time after surgery as shown in Table 3. NEAT did not change significantly between visits, please see Table 3.

### Meal associated thermogenesis (MAT)

**Fig 3:**
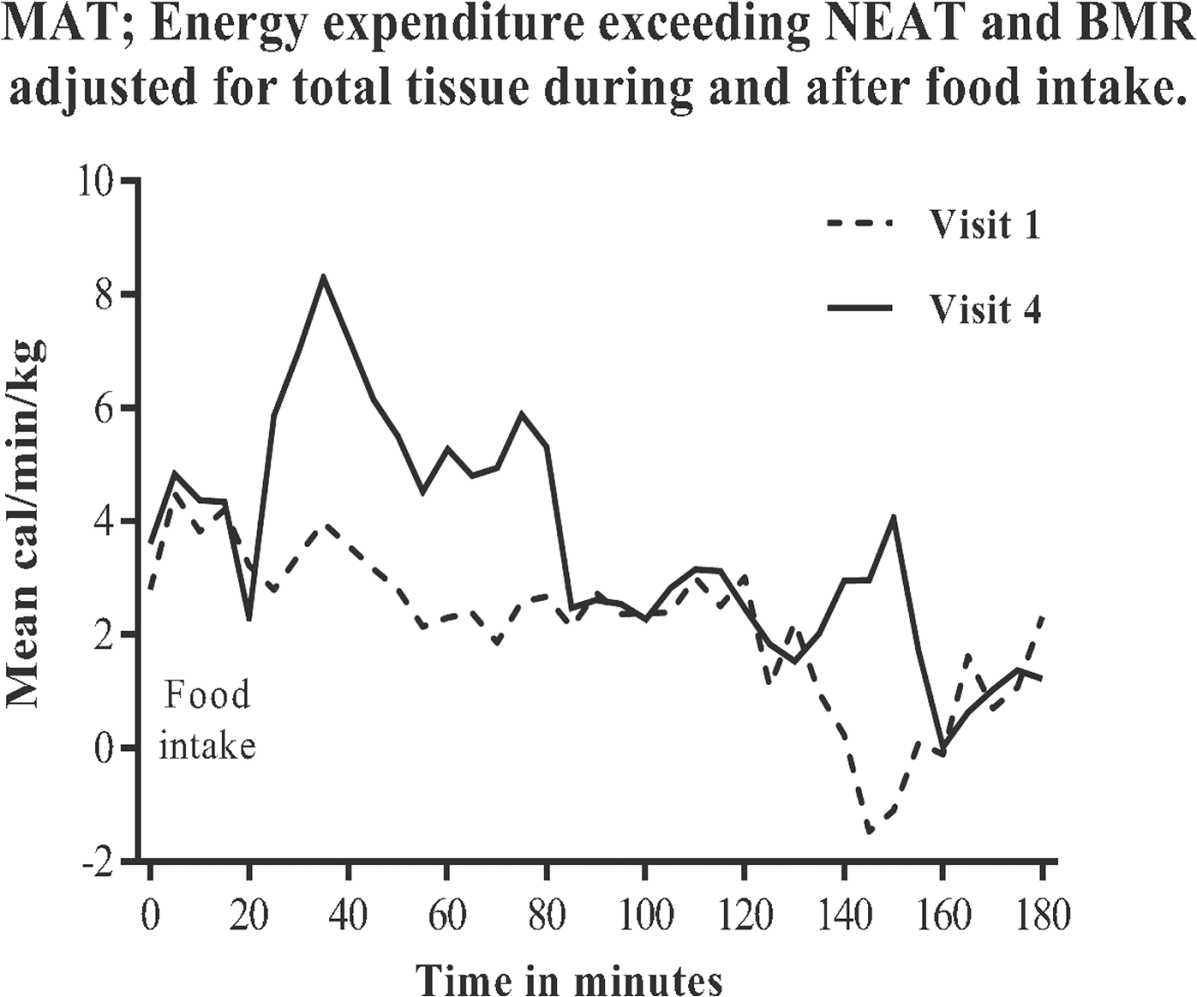
Six female subjects assessed for energy expenditure during and after a 600 kcal standard meal intake, from 18:00 to 21:00h. Time for food intake was standardized to 20 minutes. Assessments were performed before, visit 1, and at weight stability 20 months after, visit 4, gastric bypass surgery. Data are presented as MAT; energy expenditure exceeding BMR and NEAT adjusted for total tissue; mean cal/min/kg.

**Fig 4:**
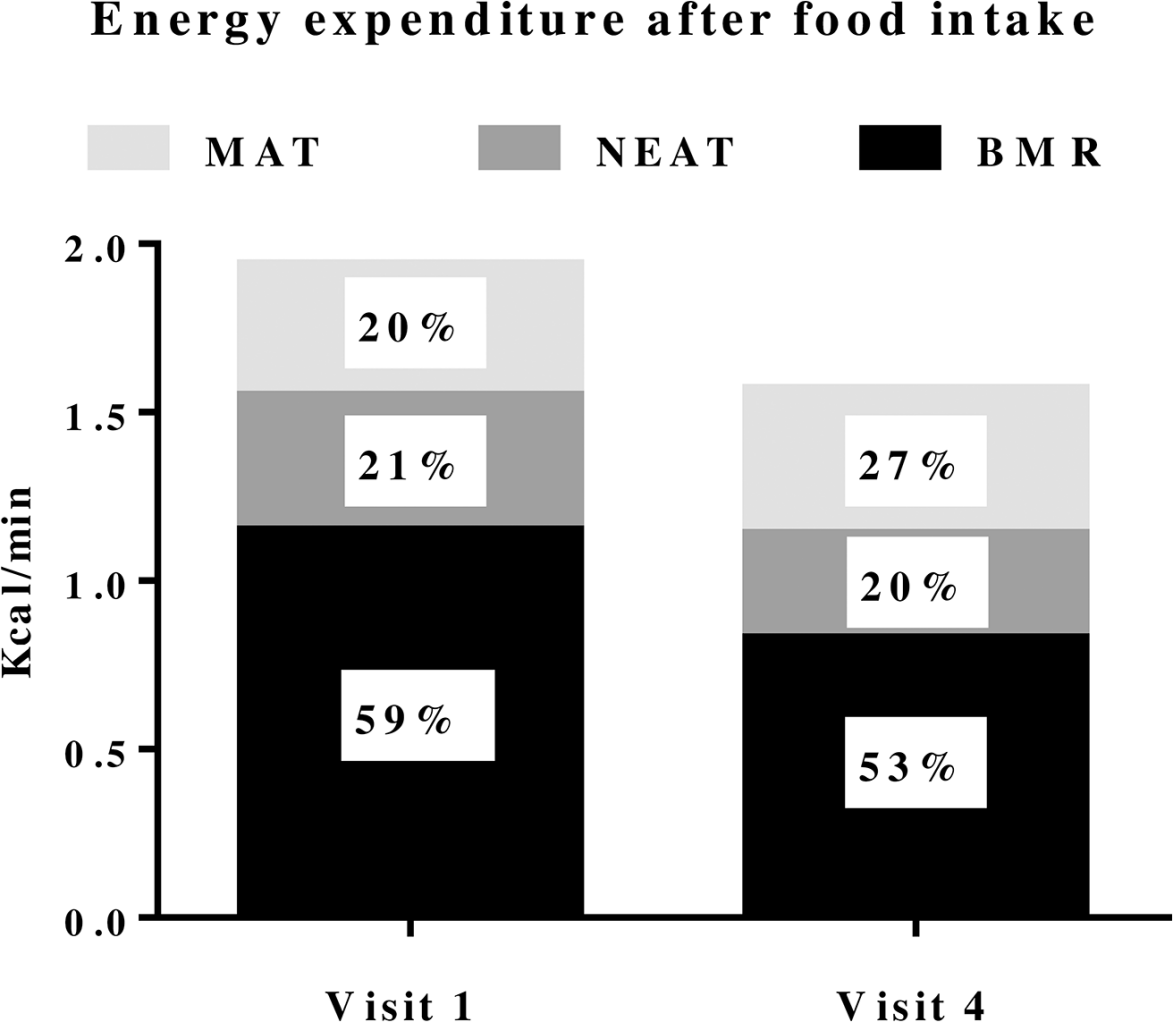
Six female subjects assessed for energy expenditure during one hour after a 600 kcal standard meal; from 18:20 to 19:20. Assessments were performed before, visit 1, and at weight stability 20 months after, visit 4, gastric bypass surgery. Data are presented as mean values in kcal/minute and as percentage of total energy expenditure.

MAT was defined as the postprandial energy expenditure exceeding pre-meal EE (*ie* BMR and NEAT). To standardise the pre-meal NEAT within the individuals only light intensity activity EE (*ie* NEAT_METS≤2_) was used in the analysis. Therefore, occasional 1 min periods with leg AU1_min_ >50 units were omitted (see Methods). Less than 12% (median 1.7%; range 0 to 11.7%) of all pre-meal NEAT recordings at each of the 24 study visits had to be removed due to spontaneous physical activity at METS>2. The resulting NEAT_METS≤2_ did not differ between study visits and are shown in Table 3. Despite the significant weight loss, MAT in absolute terms did not change between pre- and postoperative weight stability recordings (ie visit 1 and 4). However, relative to 24h EE or when adjusted for total tissue, MAT was significantly higher after surgery during the first postprandial hour as shown in Fig 3 with details given in Table 3, as well as in Fig 4.

MAT adjusted for ingested energy; MAT%, both as absolute values and adjusted for total tissue was very variable both at visit 2 and 3 but showed significantly higher values at visit 2. MAT% adjusted for total tissue was significantly increased at visit 4 compared to preoperative analyses as demonstrated in Table 3.

### Respiratory quotient (RQ)

RQ of the entire 24h period was lower immediately after surgery while patients were in a negative energy balance and losing weight (visits 2 & 3), but had increased significantly above the preoperative values at weight stability (visit 4). An identical pattern was observed for RQ during the first hour after dinner, whereas the RQ values recorded during BMR did not differ between the study visits (Table 3).

## Discussion

Meal associated thermogenesis has previously been shown to increase after RYGB in animals, but remained controversial in humans [9, 23–25]. In the present study we found that weight adjusted EE after meals increased following RYGB. Moreover the contribution of MAT as a component of total EE also increased after surgery. In absolute terms MAT did not change despite markedly reduced adipose and lean tissue mass. The physiological processes behind MAT are not completely clear but the contribution of striated muscle is minimal making adjustments of MAT for lean tissue mass not optimal. Correcting MAT for total tissue may be more appropriate as elements of the intestinal digestion/absorption and/or distant metabolic effects elicited by the gut are most probably generating MAT, thus being in the context of total tissue mass.

In the present longitudinal study we followed obese patients undergoing RYGB from before surgery, during their weight loss phase and until they reached weight stability at 18 to 22 months after surgery. The results confirm that RYGB reduced the participants’ BMI, mainly due to a loss of adipose tissue and to a lesser extent also lean tissue. As expected, whole body 24h EE decreased gradually after surgery. As EE is differentially influenced by lean mass, fat mass and visceral function and as the components of body composition changed in different amounts it required each component of EE to be analysed.

BMR decreased along with body weight as expected. The lean tissue, which is predominately muscle, has a high metabolic rate at rest and consequently determines whole body BMR. BMR when related to lean tissue was similar across visits suggesting that the basal metabolic rate of these tissues was not influenced by the surgical procedure. These results contrasts to those reported by Faria et al [26], showing increased resting metabolic rate after RYGB but are in agreement with other studies using metabolic chambers over 24h [10, 11, 27].

Repeated assessment of activity thermogenesis requires standardised study conditions [20, 21, 28–32]. Our chambered condition allowed detailed recordings of low intensity physical activity. The individual activity levels did not differ between the study visits. These results indicate that RYGB does not reduce fidgeting or other types of low intensity physical activity as would be predicted by the amount of weight loss after RYGB [33]. A moderate but significant reduction in night-time activity was apparent when the participants had again reached weight stability (visit 4). The reason to this remains to be investigated but it can be speculated that the marked weight loss relieves the load on certain body regions in lying position and in turn reduces the need for night time body movements.

It was not possible to increase the amount of food in test meals at visit 2 and 3 because of the profound impact of RYGB on the ability of patients to consume a substantial meal in the short term postoperative state. Equally it was not advisable to reduce the standard meal during visits 1 and 4 as too few calories would not generate a sufficient MAT response and allow differences to be detectable within the chamber. Meals were accordingly chosen to provoke an optimal energy metabolism and gut hormone response for minimum calories and the smallest volume of the meal. Our results in the present study are consistent with findings from our previous cross-sectional comparison performed nine years after surgery where RYGB patients had higher MAT compared to weight loss matched patients who had vertical banded gastroplasty [9]. Results from the two studies reinforce each other as the surveys were performed under equivalent protocols and in cross-sectional setting as well as in prospective conditions with included subjects as their own controls. Results indicate that energy expenditure is influenced early after gastric bypass surgery and that the pronounced metabolism remains long term postoperatively. The present results are also consistent with findings in RYGB operated rats that showed prospective increases in EE after meals which were higher than in sham operated body weight matched rats [23]. It is interesting to note that energy expenditure in rats after sleeve gastrectomy-induced weight loss did not change [34].

The changes in EE after biliopancreatic diversion are consistent with our findings after RYGB [13] and both operations share the feature of nutrients rapidly entering the jejunum (alimentary limb). The altered anatomy after RYGB changes the gastrointestinal and central neuroendocrine signalling after food ingestion, which in turn may induce the increase in energy expenditure. Postprandial release of bile acids and gut hormones such as GLP-1, glucagon and oxyntomodulin are markedly enhanced after RYGB [35] and may together and in concert influence total energy expenditure both locally in the gastrointestinal tissues and at distance, e.g. in brown adipose tissue (BAT) [36–39]. Vosselman et al have shown that glucose uptake in BAT increases after a meal in un-operated humans which indicates a potential for BAT [40]. In rats duodenal exposure to lipids activates vagal afferents which in turn activate thermogenesis in brown adipose tissue (BAT) [41]. If such a mechanism exists also in man, it may be speculated that the neural activation of BAT becomes enhanced when the nutrients instead enter the jejunum after RYGBP.

Intestinal mucosal hypertrophy and hyperplasia has been observed in rodents after RYGB [42]. Spak et al showed that the human Roux limb mucosa also has increased mitotic activity, but little or no hyperplasia. The surface epithelial area was reduced with lower and broadened villi with up-regulated cell turnover consistent with increased EE [43]. In line with this, the gut as a thermogenic organ was recently highlighted as it was shown that small bowel hypertrophy was associated with increased metabolism, which may partly explain the enhanced EE after meals [23, 44]. Saeidi et al demonstrated in rats after RYGB that the alimentary limb’s mucosal glucose metabolism increased, turning the intestine into a major organ for glucose disposal [44]. This effect was so prominent that it influenced whole-body glucose disposal with potential to improve glycaemic control in type 2 diabetes. The present results indirectly support increased postprandial glucose metabolism as the meal-associated thermogenesis was also associated with an increase in RQ.

The number of subjects in our study was limited to six. Our power calculations based on our previous data indicated that by using accurate and precise methodology such as a respiratory chamber and studying the patients over 24h would allow us to detect the differences in EE using only six patients. These patients are representative for the RYGB population based on age, gender and BMI both before and after surgery. In addition, the subjects exhibited typical biochemical improvements and characteristic changes in postprandial gut hormonal responses after RYGB. The patients and our findings also compared favourably with our previous observations in the cross sectional study performed nine years after RYGB [9]. Furthermore even with a small number of participants the effect size was significant and the data consistent with our previous observations of increased energy expenditure after food intake and during 24 hours.

We made a conscious choice in studying females only to reduce study variables and confounders such as differences in sex hormones. Females were also optimal to study as they form the major part of patients who desire and are considered for surgical obesity treatment.

Further studies are needed to be able to compare gender differences regarding energy metabolism after bariatric surgery.

By the choice of foods and standard meals we aimed at creating repeatability as well as comparability between subjects, within subjects during repeated measurements and to former studies over energy expenditure and gastrointestinal hormones. The standard semi liquid meal served in the morning (consisting of 8 E% protein, 36 E% carbohydrate and 56 E% fat) is not a typical breakfast. The fat content was also high in both the standard breakfast and dinner at visit 1 and 4. Notwithstanding, the meals were well tolerated both before and after surgery.

In conclusion: RYGB surgery was associated with decreased basal metabolic rate, unchanged non-exercise activity thermogenesis but with an increase in energy expenditure after food intake when corrected for body composition. Enhanced postprandial RQ after RYGB suggests increased glucose metabolism, presumably in the small bowel.

## Supporting Information

S1 Table Accelerometer activity units during different living activities classified as METS. (TIFF)
