# Supplementary figures and images for: Roux-en-Y Gastric Bypass Surgery Increases Respiratory Quotient and Energy Expenditure during Food Intake
Source: PLoS One. 2015 Jun 22;10(6):e0129784. doi: 10.1371/journal.pone.0129784 (PMC4476618; doi:10.1371/journal.pone.0129784)

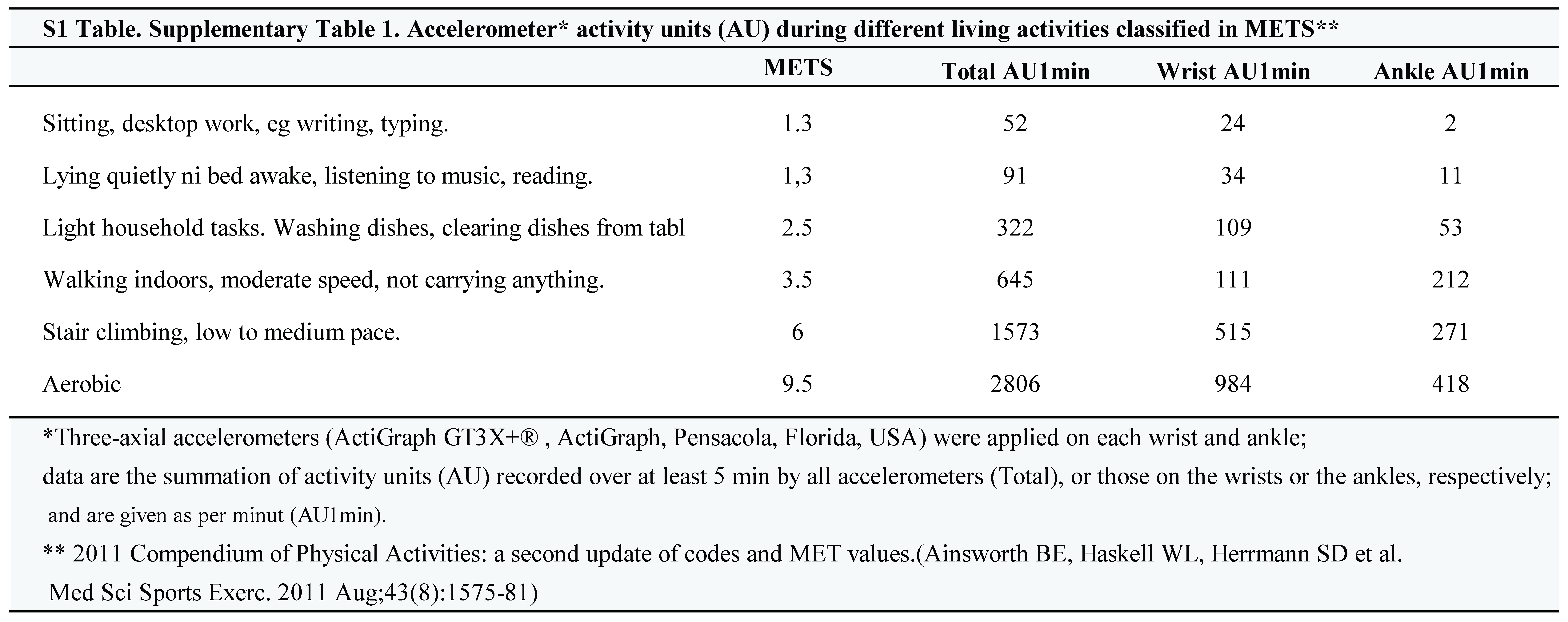

Supplement: S1 Table — (TIFF) [file pone.0129784.s001.tiff]
